# Elimination of *Escherichia coli* in Water Using Cobalt Ferrite Nanoparticles: Laboratory and Pilot Plant Experiments

**DOI:** 10.3390/ma12132103

**Published:** 2019-06-29

**Authors:** Elmer Gastelo, Juan Montes de Oca, Edward Carpio, Juan Espinoza, Pilar García, Silvia Ponce, Juan Rodriguez

**Affiliations:** 1 Center for the Development of Advanced Materials and Nanotechnology, Universidad Nacional de Ingenieria, Av. Tupac Amaru 210, Rimac, Lima 15333, Peru; 2 Istituto de Investigación Científica (I.D.I.C), Universidad de Lima, Av. Javier Prado Este 4600, Surco, Lima 15023, Peru

**Keywords:** cobalt ferrites, photocatalysis, antibacterial activity, pilot plant

## Abstract

This paper focuses on the synthesis of cobalt ferrite nanoparticles by the sol–gel method and their photocatalytic activity to eliminate bacteria in aqueous media at two different scales: in a laboratory reactor and a solar pilot plant. Cobalt ferrite nanoparticles were prepared using Co(II) and Fe(II) salts as precursors and cetyltrimethyl ammonium bromide as a surfactant. The obtained nanoparticles were characterized by X-ray diffraction, scanning and transmission electron microscopy. *Escherichia coli* (*E. coli*) strain ATCC 22922 was used as model bacteria for contact biocidal analysis carried out by disk diffusion method and photocatalysis under an ultraviolet A (UV-A) lamp for laboratory analysis and solar radiation (radiation below 350 W/m^2^ in a typical cloudy day) for the pilot plant analysis. The results showed that cobalt ferrite nanoparticles have an average diameter of (36 ± 20) nm and the X-ray diffraction pattern shows a cubic spinel structure. Using the disk diffusion technique, it was obtained inhibition zones of (17 ± 2) mm diameter. Results confirm the photocatalytic elimination of *E. coli* in water samples with remaining bacteria below 1% of the initial concentration during the experiment time (30 min for laboratory tests and 1.5 h for pilot plant tests).

## 1. Introduction

One of the most important problems in developing cities around the world is the microbiological contamination of drinking water. It is estimated that 80% of sewage water is released by industries without any further purification process. On the other hand, wastewater discharges in fresh water and coast seawater are the major sources of fecal microorganisms. As a result, there is a harmful impact on water quality with huge consequences in human health [1]. Traditional wastewater methods, such as UV radiation, and ozonolysis, are not commonly used due to the high operation costs, their low yield, and the production of intermediate species that could be more harmful for human intake [2,3].

Among wastewater purification techniques, photocatalysis has shown high yields of bacteria inactivation [4]. This technique uses absorption of light (UV and visible) by a material called photocatalyst. In photocatalysis, a semiconductor under solar radiation could promote an electron from the valence band to the conduction band, generating an electron-hole pair. These charge carriers could then drive redox reactions and possible degradation reaction of contaminants and microorganisms in water. However, the most popular photocatalyst up to now, the TiO_2_, has a wide band gap (3.03 eV rutile and 3.18 eV anatase), which is in the UV-A range and represents less than 5% of the solar spectra that get transmitted through the atmosphere [5]. Several initiatives have been implemented to develop photocatalytic materials with better absorption properties; for example, Reinosa and colleagues [6] studied composite materials based on TiO_2_ and ZnO. Nowadays, other materials, for example, ferrite nanoparticles, develop great interest because of their capability to absorb visible light, which is less energetic than UV light. 

Ferrites (MFe_2_O_4_) are magnetic materials containing ferric and M(II) ions, where M could be Co, Ni, Zn, etc. [7], and they have an inverted spinel structure. These materials have been extensively studied due to their applications in magnetic storage devices, magnetic fluids, drugs transport systems, magnetic resonance imaging, medicine, and catalysis [8,9]. Among them, cobalt ferrite presents high tensile and magnetic anisotropy, good chemical stability and hardness properties [10], and its good optical absorption in the visible range makes it suitable for photocatalytic applications. Cobalt ferrite, with a band gap of 1.3 eV [11], is one of the materials with the larger absorption in the visible range, and has been used to study the photocatalytic degradation of organic materials and bacterial inactivation [12,13]. However, according to our knowledge, there are still few works about the photocatalytic activity of these material in large scales, compared to other photocatalytic materials such as TiO_2_ and ZnO [14,15]. In this work, cobalt ferrite nanoparticles have been synthesized and used to photocatalytically disinfect water, scaling up from the laboratory to the pilot plant with promising results in the biocidal field.

## 2. Materials and Methods

### 2.1. Synthesis of Cobalt Ferrite

All reagents used in this work were analytical grade. Ultrapure water was used in the synthesis of cobalt ferrite (Milli Q, Merck Millipore, USA). The cobalt ferrite nanoparticles were obtained following the sol–gel method. Precursor solution containing 0.16 M of iron chloride tetrahydrate (FeCl_2_·4H_2_O-Sigma Aldrich-USA) and 0.08 M of cobalt nitrate hexahydrate (Co(NO_3_)_2_·6H_2_O-Sigma Aldrich-USA) was stirred in a glove box chamber filled with N_2_. After stirring, enough KNO_3_ (Sigma Aldrich-USA) and CTAB (Sigma Aldrich-USA) were added to reach a final concentration of 0.8M and 0.16%w/v, respectively. After that, a coprecipitation was promoted adding drop by drop 2.0 M potassium hydroxide (KOH-Merck-USA) until pH = 11. Then, the solid and solution were placed in a closed jar and heated at 95 °C during 16 h in an aging process for selective oxidation. After that, the system was cooled down until room temperature is reached and the solid was then washed with distilled water using cycles of centrifugation (7500 rpm) and redispersion until pH = 7.

### 2.2. Nanoparticle Characterization

#### 2.2.1. Crystalline Structure

The nanoparticle structure was analyzed using X-ray diffraction (XRD, BRUKER d8 advance, Karlsruhe Germany) using CuKα radiation (1.5418 Å) ranging 2θ angles from 20° to 70° at 40 kV. The operation temperature was kept constant at 23 °C and the operation time was 1h. No further refinement procedure was used after diffractograms were taken. 

#### 2.2.2. Morphology

SEM images were taken using a field-emission scanning electron microscopy (FE-SEM-Hitachi Regulus 8230, Tokyo, Japan) operating at 5.0 kV. Images were taken at a constant magnification of 200 kX. For that purpose, approximately 1 mg of the cobalt ferrite was dispersed in 2.5 mL of water using ultrasonic treatment in an ultrasonic water bath (Branson, Danbury, Connecticut, USA) for 10 min. After that, 100 μL of the sample was put onto silicon dishes and the system was dried in a desiccator for 1 h. 

### 2.3. Antibacterial Activity

To measure antibacterial activity, the Kirby–Bauer method was used, as described by Wikins [16]. *E. coli* were grown on an agar plate with disk-shaped filter papers coated with cobalt ferrite nanoparticles. For coating on disk-shaped filter papers, 0.05 g of dried cobalt ferrite was dispersed on 10 mL of sterile water by sonication (distilled water prepared and stored in closed bottles and sterilized at 121 °C and 6895 Pa for 20 min) and then the disk-shaped filter papers was immersed on the cobalt ferrite dispersed.

Finally, the plate was incubated for 24 h at 35 °C in a convection oven. Gray scales have been used to show the color contrast in order to distinguish between the areas of normal growth and the areas where there was no bacteria growth, named inhibition zones. The inhibition zones formed after the incubation process were measured by duplicate using a ruler. Results are expressed as mean values and standard deviations. 

### 2.4. Photocatalytic Activity

**Figure 1:**
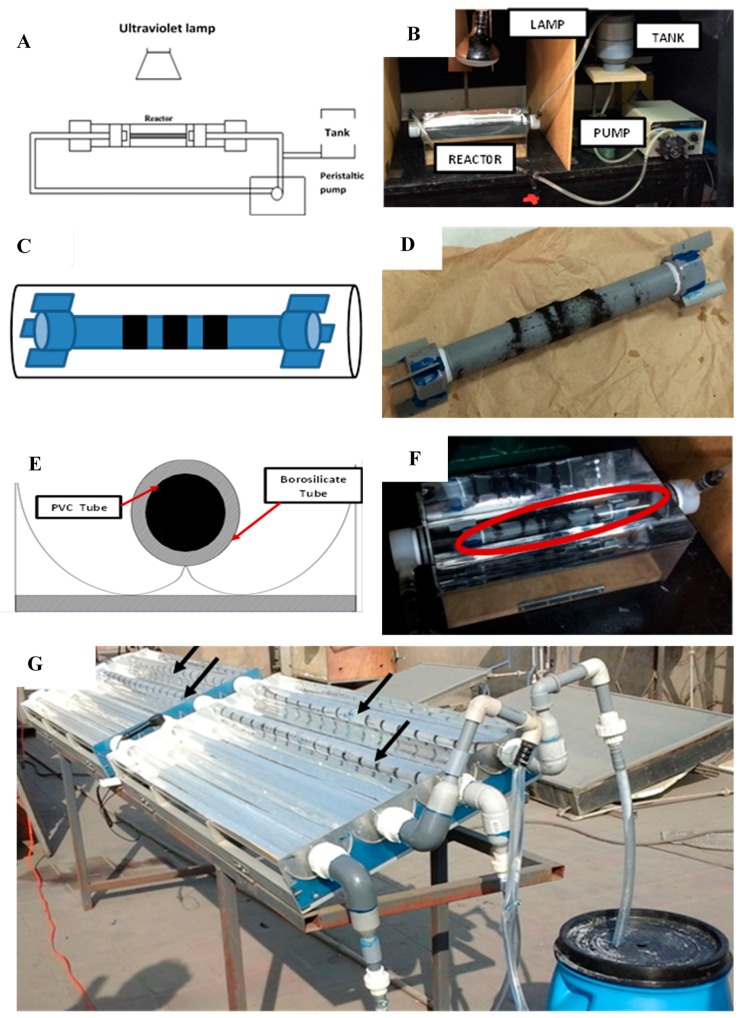
(**A**) Schematic representation of the reactor used in the laboratory scale. (**B**) Reactor used in the laboratory. (**C**) Schematic representation of cobalt ferrite nanoparticles magnetically supported on the PVC tube. (**D**) Real PVC axial tube with cobalt ferrite nanoparticles magnetically supported. (**E**) Schematic representation of inner PVC tube. (**F**) Picture of reactor used in the laboratory scale showing the cobalt ferrite nanoparticles magnetically supported on the PVC axial tube. (**G**) Solar Pilot plant during the irradiation process, the arrows show the cobalt ferrites supported on the PVC tubes.

Two devices were used to perform the photocatalytic activity of fabricated cobalt ferrites: a laboratory reactor with 1 L capacity (Figure 1A–F) and a solar pilot plant with 200 L capacity (Figure 1G).

In both analyses, an *E. coli* solution was prepared as follows; first, 0.85% *w*/*w* NaCl solution was prepared and stored in closed bottles and sterilized at 121 °C and 6895 Pa of pressure for 20 min. Culture media (Merck KGaA, Darmstadt, Germany) was dissolved using the sterilized saline solution and *E. coli* was incubated here for 12 h under continuous agitation at room temperature at pH = 6.5. After that, the solution was centrifuged at 5000 rpm for 15 min and washed with sterile saline solution.

For the laboratory analysis, 1L of synthetic water containing (7.95 × 107 ± 6.03 × 107) CFU/mL *E. coli* was placed in the laboratory device and the process was carried out for 60 min by duplicate. Samples were taken for analysis every 15 min. Samples were analyzed by successively diluting 1:10 every solution in a saline sterile solution. One milliliter of the final dilution was filtered in a sterile membrane filter for microbiology (PALL-USA, φ = 47 mm, porous size = 0.45 μm). The filter was placed onto an absorbent sterile pad (PALL-USA, φ = 47 mm) embedded in a culture broth made from lauryl sulfate solution (Oxoid- UK). Bacteria concentration was determined after an incubated process at 40 °C for 16 h. 

On the other hand, for the pilot plant experiments, the *E. coli* and NaCl solution were prepared using drinking water from the tap (pH = 7.1, conductivity = 11.62 μS) and the process was similar to the previously described. These experiments were carried out for 1.5 h taking samples every 15 min and the whole experiment was made by duplicate.

Regarding the devices, both are based in a compound parabolic collector (CPC), as described by Malato [17]. The laboratory device uses a borosilicate glass tube with 200 mm length and 50 mm diameter, and the pilot plant device uses a 1500 mm length and 50 mm tube of the same material. In both cases, polyvinyl chloride tubes (PVC tubes) containing several magnets were placed axially inside the borosilicate glass tubes. Such a configuration was used to support the cobalt ferrite and let it in contact with the water flow. The reactor at the laboratory scale was illuminated with a 220 Watts lamp (OSRAM Ultravitalux, Gutenbergstraße, Germany) and a water flow of 0.8 L/min was kept constant. At the pilot plant scale, sunlight was used during the whole experiment. The irradiance was measured using a radiometer Sper Scientific 850 009 (Sper Scientific direct, Phoenix, Arizona, USA) and a water flow of 20 L/min was kept constant. 

### 2.5. Analysis of Iron Release during Laboratory Device Operation

Iron leaking in the laboratory device during operation was determined by atomic absorption spectrometry (AAS) by AA-6701FS equipment (Shimadzu Scientific Instruments, Columbia, SC, USA); samples were taken from water pumped every hour in two sets of individual experiment. Aqueous samples undergo acid digestion with hydrochloric acid before AAS analysis. Analyses were performed in an air–acetylene flame with a lamp current of 7 mA. The total iron reported was calculated as mean value and standard deviation for each time. 

## 3. Results and Discussion

**Figure 2:**
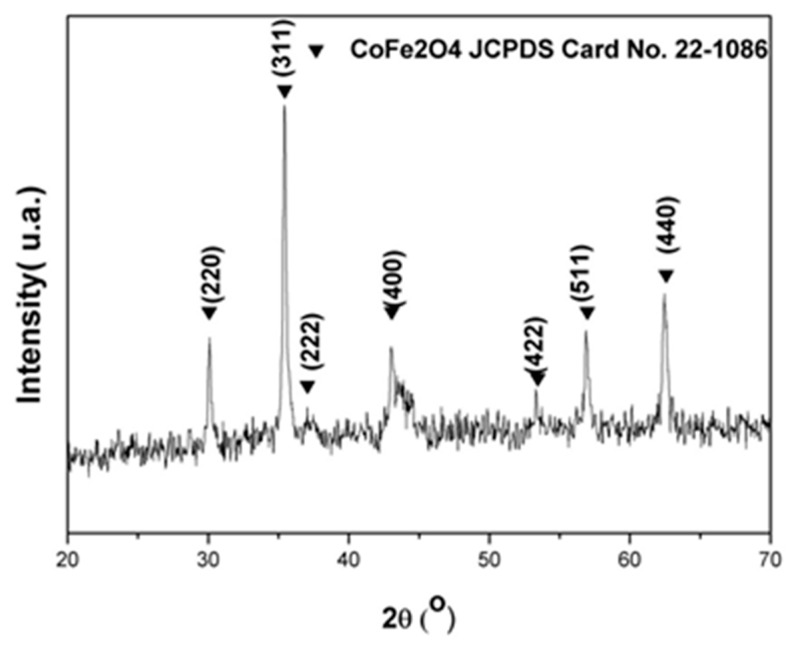
Sample diffractograms confirming the cobalt ferrite structure.

**Figure 3:**
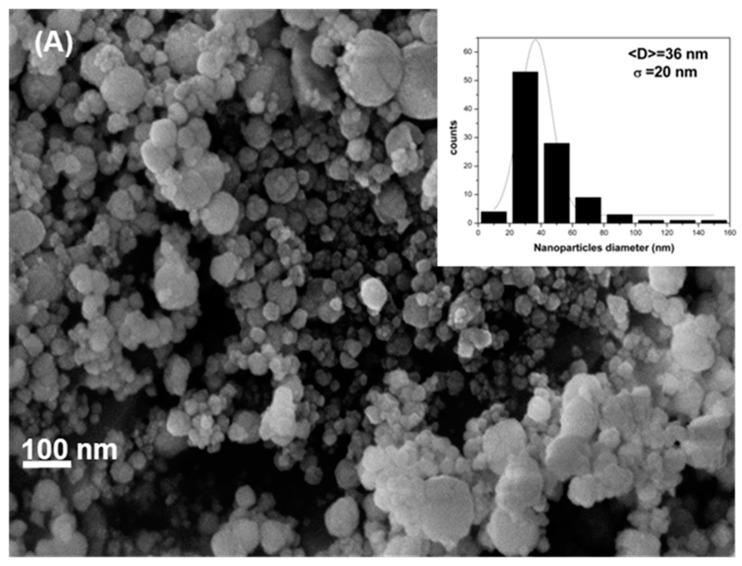
Field-emission scanning electron microscopy (FE-SEM) micrographs of cobalt ferrite nanoparticles. The inset shows the diameter distribution.

The cobalt ferrite nanoparticles were characterized by XRD to determine the crystalline structure. Figure 2 shows the crystalline plans (220), (311), (222), (400), (422), (511), and (440), which are representative for the spinel structure of cobalt ferrites, as described by Sajjia [18]. Cobalt ferrite nanoparticles aggregate when dry, as shown by the FE-SEM analysis in Figure 3. Particles are spheroids with diameter (36 ± 20) nm, calculated from pixel analysis using ImageJ v1.49 program. The addition of CTAB in the synthesis process is very important to control the nanoparticle size. As particles have negative surface charge in basic aqueous solution (isoelectric point = 6.5 [19]), electrostatic interaction between particle surface and the cationic surfactant led to the formation of a protector shell that not only prevents particle growth but also provides steric stabilization against further particle aggregation [20].

**Figure 4:**
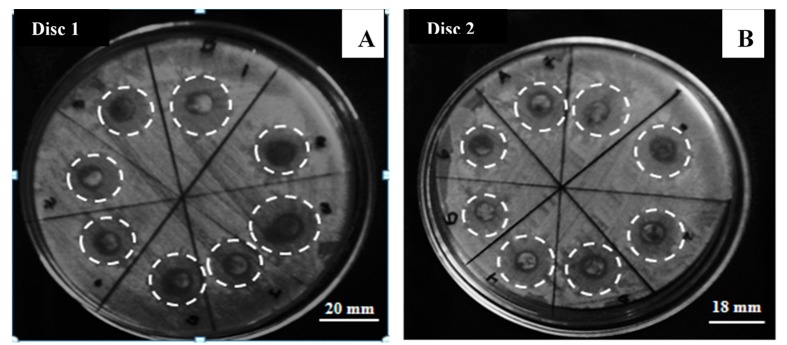
*E. coli* incubated 24 h with filter paper shaped discs coated with cobalt ferrites. Typical inhibition bacteria zones of (**A**) disc 1, and (**B**) disc 2.

As shown in Figure 4A,B, inhibition zones were formed after the incubation process and it is observed an average bacteria inhibition zone of 17.3 ± 0.2 nm, which is characteristic of a very good antibacterial material. Table 1 summarizes the measure diameters in every disk. 

Accord the cobalt ferrite dispersion change in the discs, the inhibition zones change too. The dispersion of cobalt ferrite is an important factor in the process; however, due to the magnetic properties of cobalt ferrite, the dispersion is not stable and the nanoparticles can agglomerate; this difference in inhibition zone is clearly shown in Table 1.

There are many possible mechanisms proposed to describe the biocidal activity of cobalt ferrite nanoparticles. It was suggested by Sanpo [21,22] that ferrite affects the division cycle of bacteria by attaching to the cellular membrane.

### 3.1. Photocatalytic Activity

**Figure 5:**
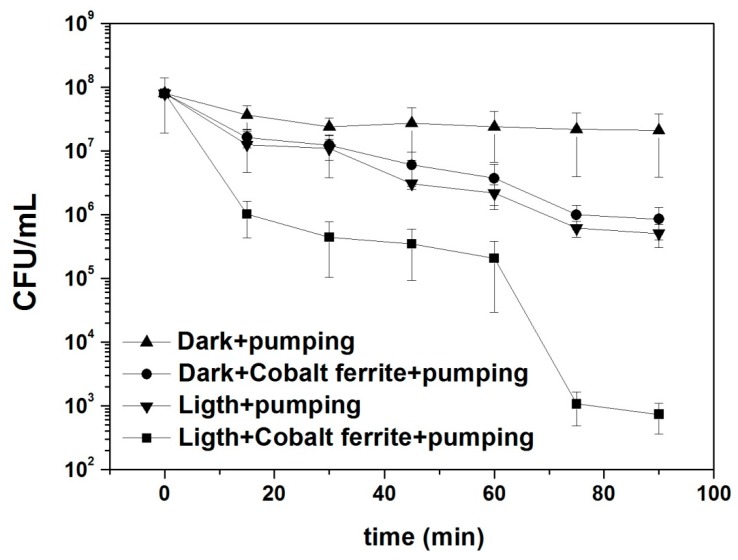
*E. coli* concentration as function of time during photocatalytic experiments using the reactor at the laboratory scale. Black triangles: dark+pumping; black circles: dark+cobalt ferrite+pumping; black inverse triangles: light+pumping (photolysis); black squares: light+cobalt ferrit+pumping (photocatalysis).

Photocatalytic experiments at the laboratory and pilot plant scale were performed as described in the experimental part. Figure 5 shows four typical experiments carried out by using the laboratory device. First of all, one experiment was carried out in the absence of light and ferrites in order to check the possible mechanical stress that can kill bacteria in the device. After 10 min of flowing, bacteria concentration remains constant at (2.36 ± 1.63) × 10^7^ CFU/mL. The second experiment was performed using cobalt ferrite nanoparticles in the absence of light and the third one, using light from the lamp without cobalt ferrite nanoparticles. Both experiments give similar results eliminating *E. coli* in water samples in about two orders of magnitude. The fourth experiment was accomplished using light and cobalt ferrite nanoparticles. In the first 30 minutes, bacteria viability reduces approximately three orders of magnitude; after that, biocidal activity increases, and at 60 min of the photocatalytic experiment, the remaining bacteria concentration tends to the limit of detection of the technique. According to scientific literature, cobalt ferrite is a semiconductor with a band gap of ~1.2–1.3 eV [11,12]. In the presence of solar radiation, this material can produce electron-hole pairs as electrons can move from the valence to the conduction band. Due to the nanometric size of the material, electrons can migrate to the surface and interact with water molecules producing free **•**OH radicals that are capable to destroy cellular membrane producing the elimination of bacteria.

**Figure 6:**
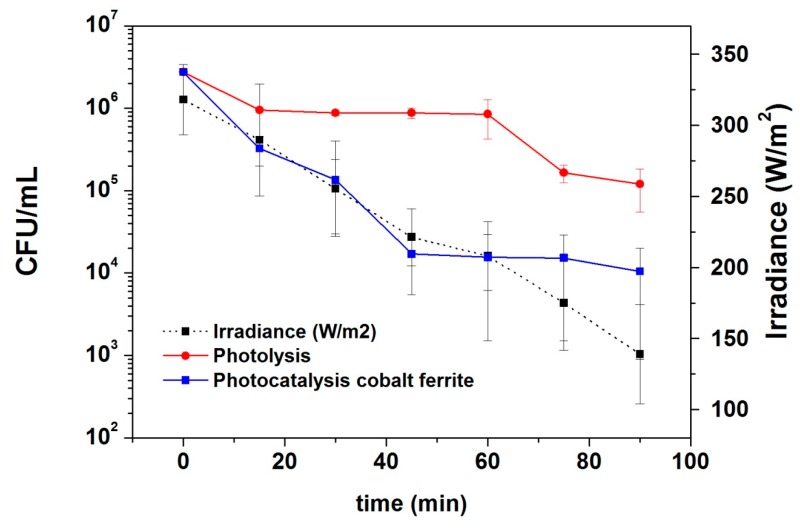
*E. coli* concentration as function of time in the pilot plant device during photocatalytic experiments. Black squares: irradiance; red circles: photolysis; blue squares: photocatalysis using cobalt ferrite nanoparticles.

Figure 6 shows the solar radiation during the experiments carried out by the pilot plant reactor in a typical cloudy day in Lima with the comparative experiment between photolysis (experiment carried out using sunlight without cobalt ferrite) and photocatalysis (using both sunlight and cobalt ferrite nanoparticles). The final concentration of *E. coli* drops to 1.2 × 10^5^ ± 6.5 × 10^4^ CFU/mL for the photolysis and 1.1 × 10^4^ ± 9.6 × 10^3^ CFU/mL for the photocatalysis, confirming the biocidal activity of cobalt ferrite nanoparticles.

#### 3.1.1. Kinetic Studies

Data from the reactor at laboratory scale and the pilot plant were fit using first order equation as suggested by Ouyang [23]. The first order equation provides the best correlation coefficient when data were fit; also, first-order kinetic mechanism is characteristic of this type of photocatalytic process. The relative amount of *E. coli* remaining in the testing solution (*N/N*_0_) decay exponentially as a function of the “*k*” factor (first-order kinetic constant), according to

(1) $$log\left(\frac{N}{N_{0}}\right)=-kt$$

where *N* is the *E. coli* concentration (CFU/mL) as a function of time and *N*_0_ is the initial *E. coli* concentration (CFU/mL); results are shown in Table 2 and Table 3. Once again, the effect of cobalt ferrite nanoparticles in the elimination of *E. coli* is noticeable, by approximately doubling the kinetic constant value.

#### 3.1.2. Analysis of Iron Release during Laboratory Device Operation

**Figure 7:**
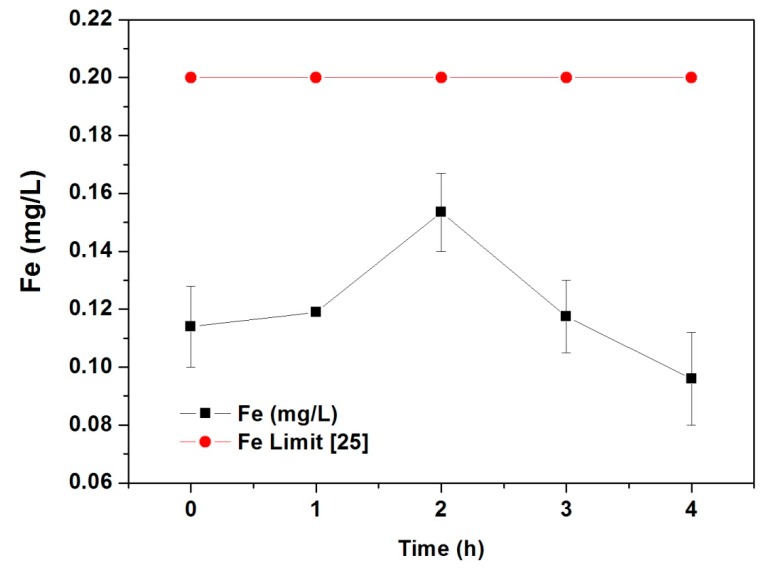
Total iron leaking in water within 4 h of pumping in the lab-scale reactor.

The total iron concentration in water pumped in the laboratory device is shown in Figure 7. According to the World Health Organization (WHO), there are no guideline values for iron concentration in tap water [24], but the European Union state a maximum iron amount of 0.2 mg/L [25]. During the first 4 h of continuous flow, the device does not release enough iron to reach the critical concentration. As the solubility of Fe(III) ions is too small in ultrapure water due to the low Kps value of Fe (III) hydroxide (Kps = 2.5 × 10^−39^) [26], the amount of iron release is mainly produced from cobalt ferrite particles and not from dissolved Fe(III). 

## 4. Conclusions

Cobalt ferrites nanoparticles were synthesized by sol–gel method. The solid structure and morphology were determined by X-ray diffraction and field-emission scanning electron microscopy, respectively. The material has a cubic inverse spinel structure with spheroidal line particles with diameters ranging (36 ± 20) nm.

Using the Kirby–Bauer method, the antibacterial activity of the ferrite was confirmed. The inhibition zones formed in the dishes have diameters in the range of (17 ± 2) mm.

In the laboratory device, cobalt ferrite was capable to inactivate *E. coli* bacteria after 60 min of continuous flow under lamp radiation. The final bacteria concentration falls in the limit of detection of the method. In the pilot plant device, the *E. coli* final concentration drops below 0.4% of the initial concentration after 1.5 h of flow under sunlight. These results show that the method can be scaled up from the laboratory to the pilot plant. The kinetic studies fit a first-order equation with a kinetic constant higher in the photocatalysis compared to the photolysis. The total release of cobalt and iron form the material to the water flowing remain under the limits allowed in the EU laws.

## Figures and Tables

**Table 1:** Average diameter of inhibition zones by Kirby–Bauer method.

Description	Disc1 (mm)	Disc2 (mm)	Mean Value (mm)
1	20	18	19 ± 1
2	15	19	17 ± 2
3	20	19	20 ± 1
4	15	16	16 ± 1
5	16	18	17 ± 1
6	16	17	17 ± 1
7	17	16	17 ± 1
8	17	15	16 ± 1

**Table 2:** Values of k for the data from the reactor at laboratory scale: dark+pumping (without the addition of cobalt ferrite nanoparticles), dark+cobalt, ferrite+pumping (with the addition of cobalt ferrite nanoparticles), light+pumping (photolysis), and light+cobalt ferrite pumping (photocatalysis). The R^2^ parameter takes into account the fit quality.

Experiment	k (min^−1^)	R^2^
Dark+pumping	0.012 ± 0.004	0.62
Dark+cobalt ferrite+pumping	0.049 ± 0.004	0.95
Ligth+pumping (Photolysis)	0.054 ± 0.005	0.95
Ligth+cobalt ferrite pumping (Photocatalysis)	0.117 ± 0.019	0.86

**Table 3:** Values of k for the data from pilot plant: photolysis (without the addition of cobalt ferrite nanoparticles) and the photocatalysis (with the addition of cobalt ferrite nanoparticles) process and the R^2^ parameter that take into account the fit quality.

Experiment	k (min^−1^)	R^2^
Photolysis	0.031 ± 0.006	0.83
Photocatalysis	0.059 ± 0.011	0.84
